# Climatic temperature and precipitation jointly influence body size in species of western rattlesnakes

**DOI:** 10.1098/rsos.240345

**Published:** 2024-08-07

**Authors:** Jesse M. Meik, Jessica A. Watson, Drew R. Schield, Blair W. Perry, Yannick Francioli, Hannah Guss, Stephen P. Mackessy, Todd A. Castoe

**Affiliations:** ^1^ Department of Biological Sciences, Tarleton State University, Stephenville, TX, USA; ^2^ Department of Ocean Engineering and Marine Sciences, Florida Institute of Technology, Melbourne, FL, USA; ^3^ Department of Biology, University of Virginia, Charlottesville, VA, USA; ^4^ School of Biological Sciences, Washington State University, Pullman, WA, USA; ^5^ Department of Biology, University of Texas at Arlington, Arlington, TX, USA; ^6^ School of Biological Sciences, University of Northern Colorado, Greeley, CO, USA

**Keywords:** Bergmann’s rule, biogeography, body size evolution, *Crotalus*, redundancy analysis, variation partitioning

## Abstract

Both the metabolic theory of ecology and dynamic energy budget theory predict that climate influences body size through its effects on first-order determinants of energetics: reactive temperatures, carbon resources and oxygen availability. Although oxygen is seldom limiting in terrestrial systems, temperature and resources vary spatially. We used redundancy analyses and variation partitioning to evaluate the influence of climatic temperature, precipitation and their seasonalities on multivariate body size across the distributions of four species of the western rattlesnake group in North America (*Crotalus pyrrhus*, *C. scutulatus*, *C. oreganus* and *C. viridis*). Most species showed a pattern of increased body size in cooler, mesic climates and decreased body size in warmer, xeric climates. Exceptions to the pattern provided additional context through climatic idiosyncrasies in the distributions of each species. For example, the general pattern of a negative influence of temperature on body size was not apparent for *C. oreganus*, which ranges across the mildest climates overall among the four species. In contrast to previous studies, we found that seasonality had negligible effects on body size. We suggest that precipitation gradients correlate positively with resource availability in driving intraspecific body size and that temperature compounds this gradient by increasing baseline metabolic demands and restricting activity in particularly warm or otherwise extreme climates.

## 1. Introduction

Despite over a century of study, little consensus has emerged on either the generality or underlying mechanisms for relationships between body size and climate. While examples of body size–climate correlations and ecogeographic ‘rules’ abound, most hypotheses are overly simplistic, not mutually exclusive in supporting evidence, and consider effects of some climatic axes in isolation of others, despite strong interdependencies [1,2]. For example, Bergmann’s rule, the tendency for individuals of a species to be larger in cooler climates, is often considered canonical, yet many studies do not support this pattern as a rule even within endotherms [3–6]. Moreover, the explanation of increased thermal inertia in larger individuals remains the most frequent explanation for Bergmann’s rule, despite having been refuted on energetics grounds over 50 years ago [4,7]. Ultimately, most ecogeographic rules as conceptualized collapse under scrutiny and body size responses across taxonomic groups seem mostly idiosyncratic [6,8–11]. Part of this problem stems from the lack of a unified framework with which to link various axes of climate with first-order drivers of growth and body size.

Both the metabolic theory of ecology and dynamic energy budget theory predict that climate influences body size directly and indirectly through its influence on first-order supports of metabolism: oxygen, organic carbon and reactive temperatures [12–14]. From this thermodynamics perspective, climatic temperatures are relevant insomuch as they determine metabolic rates and therefore the allocation of assimilated energy and materials to either maintenance or biomass production [15–17]. Thus, body size in ectotherms, in which body temperature covaries with ambient temperature, could be particularly impacted by shifts in climatic temperatures [18,19]. The influence of temperature on ectotherm body size is context dependent: warm temperatures in combination with abundant resources can promote rapid metabolism and growth, permitting wide ranges of body sizes [20], but warm temperatures in regions with limited resources, such as deserts, could favour small body size owing to both thermal metabolic acceleration and constraints of extreme temperatures on biological activity [21,22].

While temperature influences body size through effects on metabolic rates, precipitation and seasonality are likely to influence the availability of organic carbon. Precipitation correlates positively with primary productivity and increases total energy flux to all trophic levels, particularly in arid environments [23,24]. Regardless of the strength of its correlation to climate, greater resource availability increases growth and adult body size, and has been associated with body size distributions at species and assemblage levels [4,7,25]. A third climatic axis, seasonality, influences body size through resource periodicity. Large body size facilitates resistance to starvation during resource-poor conditions [26–28]. However, for this strategy, energy storage must be maximally efficient during seasonal resource pulses so that greater absolute maintenance costs of large body size are not prohibitive [22]. Otherwise, seasonality could result in shorter growth windows and maturation at smaller body size [29,30].

Here, we conducted parallel analyses of body size variation in response to climatic gradients for four species of the western rattlesnake species group [31]: the southwestern speckled rattlesnake (*Crotalus pyrrhus*), Mohave rattlesnake (*C. scutulatus*), western rattlesnake (*C. oreganus*) and prairie rattlesnake (*C. viridis*). Together, these species occupy an expansive area of western North America from southern Canada to southern Mexico, inhabiting nearly all ecological zones within the region [32]. Rattlesnakes have annual energy budgets determined by few large meals [33,34], and given the efficiency with which they assimilate energy, small differences in resource availability likely impact growth rates and body size substantially [33,35]. We show that core temperature and precipitation variables have subtle but consistent effects on body size across these species and that once corrected for spatial autocorrelation seasonality effects are negligible.

## 2. Methods

### 2.1. Dataset

**Figure 1.**
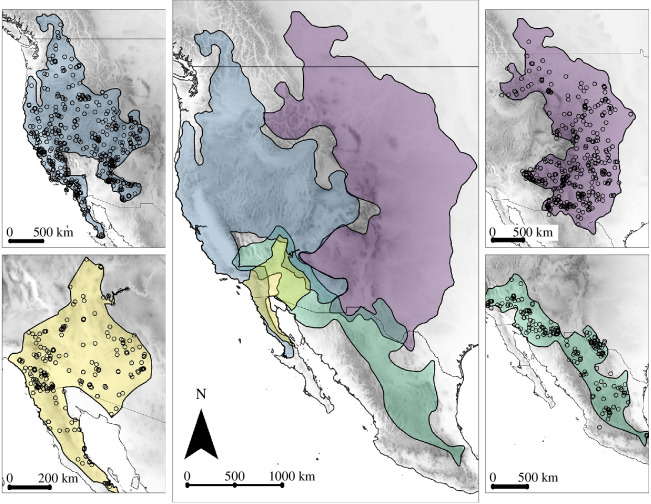
Geographic distribution of species of western rattlesnakes, clockwise from top left: *Crotalus oreganus*, *C. viridis*, *C. scutulatus* and *C. pyrrhus*. Symbols correspond to the localities of museum specimens included in this study.

We selected four species of the western rattlesnake group with collective geographic distributions encompassing western North America (figure 1). Although species-level taxonomy remains contentious for the *C. viridis* complex, we retained a conservative classification (*C. oreganus* and *C. viridis*) to facilitate comparisons with a previous study of body size variation [36], and which reflects approximately 2.8 Mya divergence [37]. We determined sex and measured a series of nine morphometric traits from 1531 subadult and adult rattlesnake specimens catalogued in natural history collections (electronic supplementary material, table S1, appendix). Assessing maturity is ambiguous without dissection, and we examined the rattle structure for indistinguishable width of successive segments, indicating asymptotic growth. The log-transformed body length distribution was nearly normal and only slightly left-skewed (electronic supplementary material, figure S1), confirming that our sample included primarily adult snakes. We confirmed taxonomic designations using locality data and phenotypic traits. Measurements were strongly intercorrelated (mean *r* = 0.84 across morphometric variables when grouping all species; electronic supplementary material, figure S1), allowing us to extract the major axis of eigenanalysis of all measurements as a composite metric of multivariate body size (MBS). Although we present total length (TL) as a metric of body size in tables and in ancillary multiple regression analyses (see below), we prefer MBS for analyses because it accounts for scaling relationships across multiple linear variables, reducing bias in any single-size surrogate. We obtained climatic data matching localities for each rattlesnake specimen from WorldClim at 2.5 arc-min resolution [38], and for analyses, we selected Bio1 (annual mean temperature), Bio4 (temperature seasonality), Bio10 (mean temperature of warmest quarter), Bio12 (annual precipitation), Bio15 (precipitation seasonality) and Bio18 (precipitation of warmest quarter). We included variables for the warmest quarter because of their correspondence to the active seasons of North American rattlesnakes. Summary data on distributional extents and climatic variables for each species are presented in table 1; data used for analyses are available from Dryad [39].

**Table 1.** Summary data (mean and s.d.) for range area (i.e. distributional extent; based on GIS layers that appear in figure 1), total length and climatic variables extracted from locality-matched specimen data for each species. WQ = warmest quarter.

	*C. pyrrhus* (*n* = 227)	*C. scutulatus* (*n* = 231)	*C. oreganus* (*n* = 609)	*C. viridis* (*n* = 465)
area (km^2^ × 10^6^)	0.2	0.9	1.5	2.1
total length (mm)	782 ± 151	754 ± 152	773 ± 153	784 ± 153
bio 1 annual mean temp. (°C)	18.5 ± 3.1	17.5 ± 2.3	11.7 ± 3.6	11.3 ± 3.6
bio 4 temp. seas. (sd × 100)	677 ± 135	636 ± 179	724 ± 176	887 ± 105
bio 10 mean temp WQ (°C)	27.1 ± 3.8	25.2 ± 3.7	20.9 ± 3.1	22.3 ± 3.2
bio 12 precipitation (mm)	267 ± 150	352 ± 129	412 ± 241	387 ± 108
bio 15 precip. seas. (cv)	63 ± 16	75 ± 20	53 ± 26	58 ± 14
bio 18 precip. WQ (mm)	45 ± 29	147 ± 72	61 ± 52	150 ± 45

### 2.2. Statistical analyses

To evaluate climatic effects on MBS for each species, we used a series of redundancy analyses (RDA) and partial RDAs (pRDA) [40,41], followed by variation partitioning [42]. RDA is a canonical extension of multiple regression to include matrices of response variables and is often used to evaluate associations between phenotypic, environmental and spatial datasets [43–45]. Linear measurements of rattlesnake specimens represented the response matrix, and climatic and spatial data alternately served as explanatory and conditioning matrices for model sets (electronic supplementary material, Extended Methods). To extract individual components of variation, we initially performed five model sets for each species (table 2); however, variance inflation factors of >10 indicated strong collinearity between measures of seasonality and the core climatic variables from which they were derived. For this reason, we performed two series of RDAs for each species: one including core climatic variables and another including only seasonality variables. Prior to analyses, we centred and scaled climatic data and log-transformed linear measurements. To account for spatial structure in an RDA table format, we vectorized raw distance matrices for each species using principal coordinates of neighbour matrices, which decomposes truncated distance matrices into new sets of orthogonal spatial variables [46,47]. Because dimorphism was similar across taxa (females were 83, 87, 86 and 93% of the TL of males for *C. pyrrhus*, *C. scutulatus*, *C. oreganus* and *C. viridis*, respectively) and to maximize power, we combined sexes for analyses of each species. We further justify this because sample sizes were skewed toward males for all species and responses were expected to be unidirectional. We partitioned variation in MBS using parameter estimates from the separate RDA and pRDA models for each species such that (i) is the amount of variation explained by climate only, (ii) is shared by climate and geographic distance, (iii) is explained by geographic distance only, and (iv) is the residual unexplained variation [42].

**Table 2.** Partitions of variation (adjusted fractions) of multivariate body size (MBS; response matrix) between climate and geographic distance (explanatory matrices) from results of RDA and pRDA for each species. Simulated *p* values are for specified models, not factor loadings, of RDAs and pRDAs of the influences of climate and geographic distance (each model was significant for only the first axis).

	*C. pyrrhus*	*C. scutulatus*	*C. oreganus*	*C. viridis*
model	Adj *R^2^*	Adj *R^2^*	Adj *R^2^*	Adj *R^2^*
core climatic variables (Clim): Bio1, Bio10, Bio12, Bio18				
MBS~Clim	0.14^c^	0.08^c^	0.08^c^	0.12^c^
MBS~Dist	0.18^c^	0.10^c^	0.19^c^	0.20^c^
MBS~Clim + Dist	0.21	0.15	0.23	0.22
MBS~Clim | Dist	0.03^a^	0.05^b^	0.04^c^	0.02^b^
MBS~Dist | Clim	0.07^b^	0.07^c^	0.15^c^	0.09^c^
shared	0.11	0.03	0.04	0.11
unexplained	0.79	0.85	0.77	0.78
seasonality (Seas): Bio4, Bio15				
MBS~Seas	0.002^NS^	0.02^a^	0.06^c^	0.05^c^
MBS~Dist	–	0.10^c^	0.19^c^	0.20^c^
MBS~Seas + Dist	–	0.13	0.21	0.20
MBS~Seas | Dist	–	0.02^a^	0.02^c^	<0.001^NS^
MBS~Dist | Seas	–	0.11^c^	0.16^c^	0.15^c^
shared	–	0	0.03	0.05
unexplained	–	0.87	0.79	0.80

^a^ *p* < 0.05.

^b^ *p* < 0.01.

^c^ *p* < 0.001.

NS, not significant.

We further examined variable loadings for pRDA models of the effects of core climate and seasonality on MBS after conditioning for the effects of spatial autocorrelation. For these pRDAs, we standardized axes to be positively correlated to the main axis of MBS variation when necessary and tested the significance of global models, axes and climatic descriptors using *F*-tests generated from random permutations of the table data [48]. To provide additional insight into body size variation across species and to compare the results of RDA with more traditional approaches, we also performed multiple linear regression analyses of centred and scaled climatic variables on log-transformed TL, without accounting for spatial effects. These models are analogous to the MBS~Clim and MBS~Seas RDA models in table 2. As with RDA, we performed two models for each species, one including core climatic variables (Bio1, Bio10, Bio12 and Bio18) and the other including only seasonality variables (Bio4 and Bio15). All analyses and data visualization were performed in R base [49], ggplot2 [50] and vegan [51]. Distribution maps were generated in ArcGIS Pro [52].

## 3. Results

All significant RDA and pRDA models were themselves significant for only the first canonical axes, simplifying interpretation to the primary gradients of variation for each species. Together, core climatic variables and spatial distance explained between 15 and 23% of the variation in MBS across species (table 2); however, most of this additive variation was driven by the spatial component. Nonetheless, after partialling out the effects of spatial autocorrelation, models of core climatic variables remained significant for all species, albeit with limited explanatory power. Shared variation was high (11%) for both *C. pyrrhus* and *C. viridis*, indicating high redundancy between climate and spatial distance in structuring MBS for these species. With respect to the effects of seasonality, pRDA models were significant for only *C. scutulatus* and *C. oreganus*, and explanatory power was even less than for core climatic variables.

**Figure 2.**
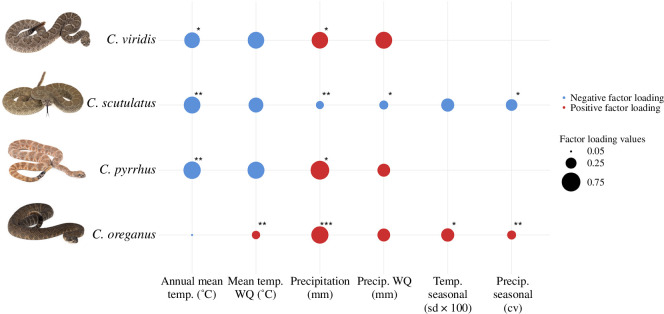
Factor loadings of the first axis from partial redundancy analyses (pRDAs) of the effects of core climatic variables and seasonalities on multivariate body size (MBS) conditioned on geographic distance for rattlesnake species included in this study; nonsignificant models excluded (i.e. seasonality models for *Crotalus viridis* and *C. pyrrhus*). All pRDAs are standardized so that positive values (red bubbles) reflect increases in MBS. Asterisks indicate climate variables that are significantly aligned with the first pRDA axis based on data permutations (only axis 1 was significant for all models): *p < 0.05; **p < 0.01; ***p < 0.001.

Partial RDA models of the effects of climate conditioned on spatial distance demonstrated that overall, temperature and precipitation influenced MBS in opposing directions (figure 2; electronic supplementary material, tables S2 and S3). For *C. pyrrhus*, *C. scutulatus* and *C. viridis*, Bio1 had significant negative loadings whereas Bio10 had a subtle, but significant positive impact on MBS for *C. oreganus*. Bio12 had a significant positive influence on MBS for *C. pyrrhus*, *C. oreganus* and *C. viridis*. Although loadings were negative for both Bio1 and Bio12 for *C. scutulatus*, the relative magnitude of vectors indicated that the general pattern of smaller body size in warmer climates was weakly supported. Partial RDAs of seasonality, significant only for *C. scutulatus* and *C. oreganus*, mirrored results from core climatic variables, indicating subtle effects at least partly confounded by strong correlations between seasonality and core climatic variables.

Overall, results from multiple regression of climate and seasonality variables on TL were broadly congruent with analogous models from canonical analyses on MBS (electronic supplementary material, table S4). Total variation explained was similar or identical across models with two exceptions: (i) RDA explained 8% of total variation in core climate for *C. oreganus* but only 4% using multiple regression and (ii) RDA explained 6% of total variation in seasonality for *C. oreganus* but only 0.7% using multiple regression. Although mostly congruent, subtle discrepancies between methods were also apparent in the effects of individual climatic variables. In our multiple regression models, the negative effects of Bio1 on *C. pyrrhus* and *C. scutulatus* TL were evident, whereas the effects of Bio12 were nonsignificant. For *C. viridis*, positive precipitation effects on TL were attributed to Bio18, whereas Bio12 was nonsignificant—in RDAs, both Bio12 and Bio18 had large positive factor loadings, but only Bio12 was significantly aligned with the first RDA axis. In contrast to RDA, multiple regression indicated nonsignificant seasonality effects on body size for both *C. scutulatus* and *C. oreganus*. Seasonality effects were apparent for *C. viridis* from multiple regression, and although these results are congruent with RDA models, partialling out spatial effects in pRDA revealed that this component of variation was confounded by spatial autocorrelation.

## 4. Discussion

Our results indicate a subtle, but consistent pattern across species where individuals throughout their respective ranges were larger in cooler, wetter environments and smaller in warmer, xeric environments. In contrast, seasonality effects were minimal and related mostly to correlation with primary climatic variables. Large body size in parts of the range with the greatest annual precipitation is consistent with indirect impacts of precipitation on resource availability. The links between resource availability, growth and adult size in snakes are well established from both supplemental feeding experiments [53,54] and observational studies correlating body size with prey size and availability [55–57]. It follows that, at broad scales, increased annual precipitation would support larger and more stable prey bases, which in turn could influence geographic variation in population densities and adult body sizes of predators. This pattern is congruent with a study that reported larger body size in western diamondback rattlesnakes (*Crotalus atrox*) from cooler, wetter regions of Arizona [58] and an interpopulation comparison of rock rattlesnakes (*Crotalus lepidus*) in which snakes were larger at a cooler, high elevation site than at a warmer, low elevation site [21,59].

Climatic effects on body size could be neutral or weakly positive in benign environments but exert stronger negative influence under conditions of extreme thermal, resource, or hydric stress, such as warm deserts, and therefore gradients that span particularly stressful conditions could have amplified climatic effects. Consideration of patterns across species seems to support this premise. *Crotalus oreganus* occupies relatively mild conditions with the coolest summer temperatures and was the only species that did not show a negative relationship between MBS and annual mean temperature (Bio1), but rather a positive influence of mean temperature of the warmest quarter (Bio10). Given the overall mild climates occupied by *C. oreganus*, this deviation from the typical pattern of strong negative effects of annual temperature on MBS might be expected. At the other end of the spectrum, climatic effects were greatest for *C. pyrrhus*, which occupies the harshest climates in terms of high temperature and low precipitation. The species attains large body sizes in comparatively mesic coastal chaparral habitats and is dwarfed in the Lower Colorado region of the Sonoran Desert. Interestingly, in this extreme climate *C. pyrrhus* is more arboreal and consumes a greater proportion of birds [60], which alludes to diet shifts under limited resources and/or behavioural mediation of direct thermal stress. The multiple regression model for *C. pyrrhus* further supports this perspective because negative temperature effects on body size were strong and countered the apparent positive influence of precipitation variables, which were nonsignificant for these models. This relationship was also inferred for *C. scutulatus*, which occupies similarly extreme, warm desert conditions.

In contrast to our analyses, Ashton [36] reported that seasonality was the main correlate of body size variation in *C. viridis* and *C. oreganus*, although the former species was larger in cooler, more seasonal climates, while the latter was smaller. Discrepancies could be partly methodological, as our ability to discern a more parsimonious pattern (i.e. both species subject to similar opposing joint effects of precipitation and temperature on body size) resulted partly from incorporating spatial autocorrelation rather than imposing phylogenetic structure in analyses of these species. *Crotalus viridis* is largest in the northern Great Plains where the active season is comparatively short and temperature seasonality is high; however, summer precipitation is also high, likely supporting a large prey base. Accordingly, *C. viridis* in Saskatchewan has high summer growth rates of juveniles [61], which could increase survivorship during brumation, where size-dependent overwinter survivorship is more closely tied to juveniles than to adults [62,63]. Consistent with this hypothesis, multiple regression analyses indicated that positive precipitation effects on body size shifted from annual precipitation (Bio12), as in *C. oreganus* and *C. pyrrhus*, to precipitation of the warmest quarter (Bio18) for *C. viridis*. Unlike *C. viridis*, *C. oreganus* reaches the largest body sizes in its southern range where annual precipitation is moderately high, precipitation seasonality is high, and summer heat is mitigated by coastal effects. Importantly, active seasons are long, permitting prolonged foraging and growth.

Of the four species evaluated, effects of precipitation were weakest for *C. scutulatus*. We suspect this equivocal influence resulted from sample size being heavily biased toward semiarid grasslands near the species median for climatic conditions followed by Chihuahuan and Sonoran deserts but with few specimens available from cooler and more mesic matorral in the southern part of the distribution. Both RDA and multiple regression were comparable for the negative influence of temperature and minimal influence of precipitation on body size for *C. scutulatus*, suggesting similar outcomes from either method despite the sampling bias. Overall, both RDA on matrices of morphometric variables (MBS) and multiple regression on TL across species resulted in congruent inferences, validating the use of RDA as well as MBS as a metric of body size. Although there were minor discrepancies in results between methods, there did not seem to be a consistent bias in one method relative to the other. For example, more variation in the body size of *C. viridis* was explained by core climatic variables in multiple regression than by RDA; in contrast, more variation in the body size of *C. oreganus* was explained by these same variables in RDA than by multiple regression. Differences mostly reflected the influence of seasonality (highlighting the ambiguity of seasonality effects on body size) and for two species multiple regression provided a relatively stronger signal for the effects of annual mean temperature over precipitation. While RDA generates composite variables by extracting information from the correlation structure (or ‘redundancy’) in a data matrix, multiple regression calculates partial regression coefficients for explanatory variables; thus, each coefficient reflects the contribution of each variable while holding the effects of other variables constant. Although multiple regression indicated a nonsignificant effect of precipitation for *C. pyrrhus*, a scatterplot of TL against annual precipitation shows a clear positive influence (electronic supplementary material, figure S2), suggesting that stronger temperature effects might have diluted weaker precipitation effects in multiple regressions of this species.

Because body mass itself is the single largest contributor to individual energetic demands, direct and indirect climate effects on energy allocation are small relative to the effects of food availability. The expected subtle and context-dependent effects of climate on body size suggest that at macroscales ecogeographic ‘rules’ are doomed to have limited explanatory power [1]. Collection periods of specimens accessioned in natural history repositories often span well over a century, and the effects of climate change on morphology will further increase the noise-to-signal ratio of analyses of body size change unless these effects are explicitly addressed. Although larger body size in cooler climates could be interpreted as a ‘Bergmannian’ cline, a nuanced perspective suggests that cooler temperatures are associated with higher precipitation, leading to greater primary productivity, at least in the context of temperate North America (e.g. [64]). In species of western rattlesnakes, the gradient of increasing body size from warmer, xeric environments to cooler, mesic environments likely results from joint effects of temperature on metabolic energy demands and restricting foraging activity and precipitation on underlying resource availability. Our ability to detect statistical signals of these direct and indirect climatic effects from specimen-level data rather than the typical practice of aggregating data into population-level summaries was enhanced by the energetic efficiency of rattlesnakes, which are responsive to small differences in prey availability [33,34]. For future efforts to understand climate–body size associations, we first recommend studies including large intraspecific sample sizes as done here, and for example [65], rather than at macroecological scales, where idiosyncratic responses and generally small intraspecific sample sizes lack the power to dissect direct and indirect factors that likely have low effect sizes.

## Data Availability

Data are available from the Dryad Digital Repository [39]. Supplementary material is available online [66].
